# Latin-American Registry of Cardiovascular Disease and COVID-19: Final Results

**DOI:** 10.5334/gh.1272

**Published:** 2023-11-01

**Authors:** Juan Esteban Gomez-Mesa, Stephania Galindo, Manuela Escalante-Forero, Yorlany Rodas, Andrea Valencia, Eduardo Perna, Alexander Romero, Iván Mendoza, Fernando Wyss, José Luis Barisani, Mario Speranza, Walter Alarco, Noel Alberto Flórez

**Affiliations:** 1 Fundación Valle del Lili, Cali, CO; 2 Instituto de Cardiología J.F Cabral, Corrientes, AR; 3 Hospital Santo Tomas, Ciudad de Panama, PA; 4 Universidad Central de Venezuela, Caracas, VE; 5 Servicios y Tecnología Cardiovascular de Guatemala S.A –Cardiosolutions, Ciudad de Guatemala, GT; 6 Clínica Adventista Belgrano, Buenos Aires, AR; 7 Hospital Clínica Bíblica, San Jose, CR; 8 Instituto Nacional Cardiovascular INCOR ESSALUD, Lima, PE

**Keywords:** Coronavirus, COVID-19, Latin America, Caribbean, SARS-CoV-2, Heart Failure

## Abstract

**Background:**

Socioeconomic factors contribute to a more severe impact of COVID-19 in Latin American and Caribbean (LA&C) countries than in developed countries. Patients with a severe or critical illness can develop respiratory and cardiovascular complications.

**Objective:**

To describe a LA&C population with COVID-19 to provide information related to this disease, in-hospital cardiovascular complications, and in-hospital mortality.

**Methods:**

The CARDIO COVID-19–20 Registry is an observational, multicenter, prospective, and hospital-based registry of patients with confirmed COVID-19 infection that required in-hospital treatment in LA&C. Enrollment of patients started on May 01, 2020, and ended on June 30, 2021.

**Results:**

The CARDIO COVID-19–20 Registry included 3260 patients from 44 institutions of 14 LA&C countries. 63.2% patients were male and median age was 61.0 years old. Most common comorbidities were overweight/obesity (49.7%), hypertension (49.0%), and diabetes mellitus (26.7%). Most frequent cardiovascular complications during hospitalization or reported at discharge were cardiac arrhythmia (9.1%), decompensated heart failure (8.5%), and pulmonary embolism (3.9%). The number of patients admitted to the Intensive Care Unit (ICU) was 1745 (53.5%), and median length of their stay at the ICU was 10.0 days. Support required in ICU included invasive mechanical ventilation (34.2%), vasopressors (27.6%), inotropics (10.3%), and vasodilators (3.7%). Rehospitalization after 30-day post discharge was 7.3%. In-hospital mortality and 30-day post discharge were 25.5% and 2.6%, respectively.

**Conclusions:**

According to our findings, more than half of the LA&C population with COVID-19 assessed required management in ICU, with higher requirement of invasive mechanical ventilation and vasoactive support, resulting in a high in-hospital mortality and a considerable high 30-day post discharge rehospitalization and mortality.

## Background

The Severe Acute Respiratory Syndrome Coronavirus 2 (SARS-CoV-2) can spread through respiratory droplets (it can stay contagious and can be suspended in the air for up to three hours), human-to-human transmission, by contaminated objects, and airborne contagion. The reported contagion rates from a patient with symptomatic infection vary by location and efficiency of infection control measures [1,2,3,4].

The coronavirus disease 2019 (COVID-19) pandemic is caused by SARS-CoV-2, an RNA virus that turned into a major public health concern after the outbreak of the Middle East Respiratory Syndrome-CoV (MERS-CoV) and severe acute respiratory syndrome-CoV (SARS-CoV) in 2002 and 2012, respectively. In total, seven human coronaviruses (HCoVs) have now been discovered, but the definitive origin of SARS-CoV-2 remains undetermined [5,6].

The COVID-19 pandemic started in Wuhan, China in late 2019; the epicenter moved across the world, then arrived at Latin America and Caribbean (LA&C) where the first case of COVID-19 was confirmed on February 25, 2020. According to the World Health Organization [7], until February of 2023 more than 753 million cases have been reported worldwide, 188 million of them from the Americas. Some of the most affected LA&C countries are Brazil (36.7 million), Argentina (10.0 million), Mexico (7.3 million), and Colombia (6.3 million). Socioeconomic factors contribute to a more severe impact of COVID-19 in this region than in developed countries; moreover, some of the comorbidities associated with a worse prognosis of COVID-19, such as obesity, diabetes, and hypertension, are highly prevalent in LA&C [8,9,10,11,12,13]. LA&C has low gross domestic product (GDP) expended in healthcare (7.9%) compared to Europe (9.8%) or North America (16.4%). Moreover, there are other factors that impact COVID-19 severity such as poor government support, fragile healthcare system, pandemic preparedness, and socioeconomic inequalities [14].

Based on the above, the CARDIOCOVID-19–20 Registry collected information of hospitalized patients with COVID-19 from 14 LA&C countries, this article aimed to describe demographics, clinical risk factors, laboratory findings, clinical presentation on admission, and clinical outcomes at discharge and 30-day post-discharge, of this population.

## Methods and Design

The CARDIO COVID-19–20 Registry is an observational, multicenter, prospective, and hospital-based registry that included patients with confirmed COVID-19 that required in-hospital treatment in LA&C institutions [15]. Enrollment of patients started on May 2020 and ended in June 2021. The Biomedical Research Ethics Committee of the Fundación Valle del Lili in Cali, Colombia approved this study under act number 409–2021. Informed consent was not required because of the observational design. A 30-day follow-up after hospital discharge was planned for every recruited patient.

The following countries participated in the CARDIO COVID-19-20 Registry (in alphabetical order): Argentina, Brazil, Chile, Colombia, Costa Rica, Ecuador, El Salvador, Guatemala, Mexico, Panama, Paraguay, Peru, Dominican Republic, and Venezuela.

Inclusion criteria to participate in CARDIO COVID-19–20 Registry included patients older than 18 years with a serological confirmed diagnosis of COVID-19 according to guidelines given by the World Health Organization (WHO) and according to each institutional and/or local guideline, who required in-hospital management for more than 24 hours, or who died during the first 24 hours after hospital admission.

The data were collected in the electronic database system REDCap [16,17]. This database system included 277 variables, which covered patient demographics, socioeconomic characteristics, medical history (CVD and non-CVD related), clinical signs and symptoms on admission, laboratory results, clinical outcomes reported at discharge, and 30-day follow-up data. The confidentiality of patient data was assured, each institution was coded, as well as the identity of every enrolled patient.

### Data Analysis

Demographics and clinical characterization of the in-hospital population was carried out through a univariate analysis. Variables with normal distribution were identified with a Shapiro Wilk test. Categorical variables are reported as a number and percentage, normally distributed continuous variables as mean with Standard Deviation (SD), and variables with skewed distribution as median with Interquartile Range (IQR). Clinical variables analyzed included laboratory tests (at admission and at discharge), pathological history, cardiovascular treatment prior to admission, clinical manifestations at admission, clinical findings at admission, cardiovascular complications during hospitalization, cardiac imaging tests, cardiovascular procedures performed during hospitalization, medication used for COVID-19, ICU admission, in-hospital deaths and post-discharge 30-day deaths. We defined the type of infection as “circulation” when the patient did not know where the infection was acquired, as “contact” when the patient was in close contact with someone with COVID-19 and knew who infected them, and as “imported” when the patient acquired the infection in a foreign country and then traveled back to his/her country. All analyses were performed using the Stata 17.0 MP version.

## Results

**Figure 1:**
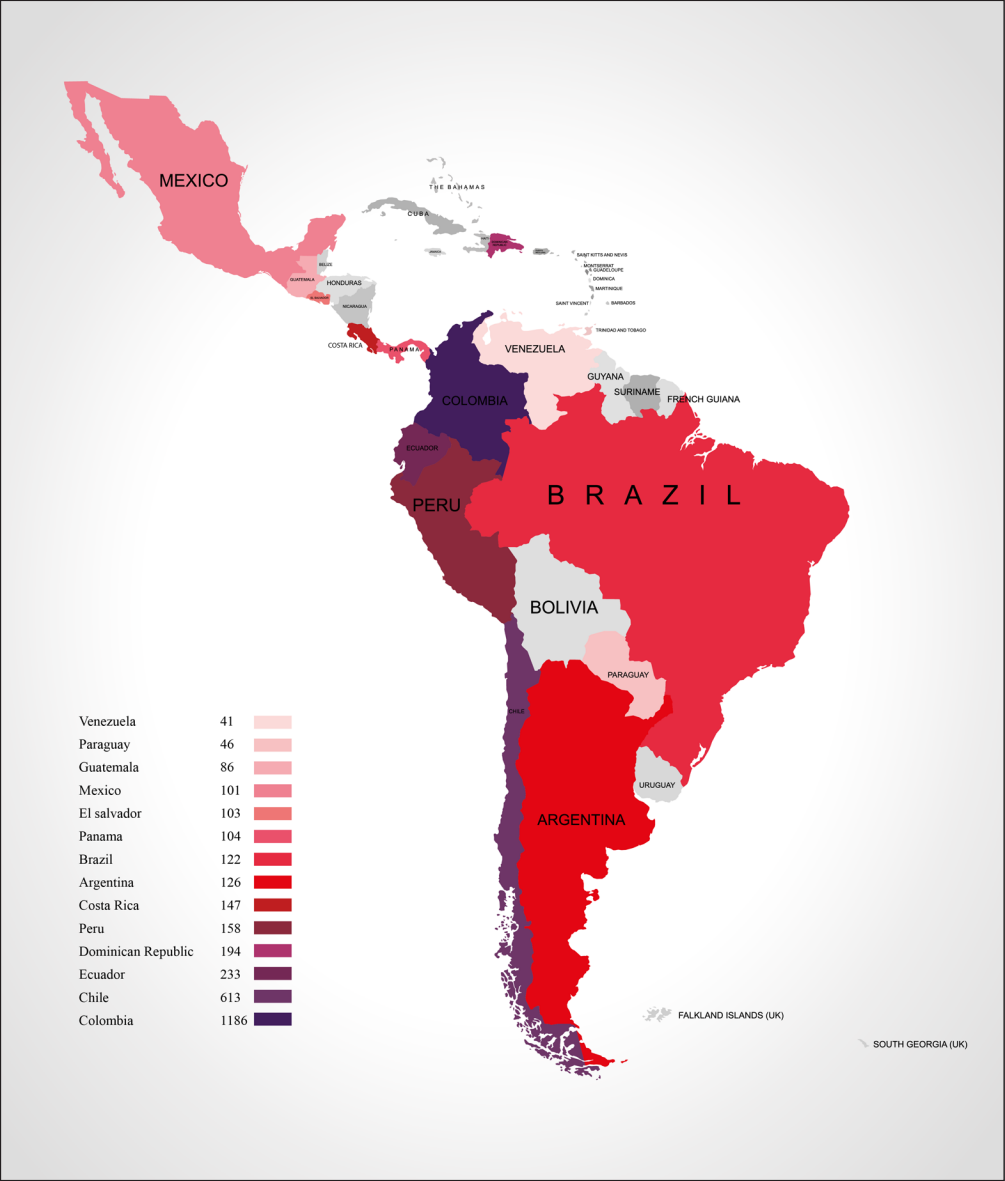
Number of patients by participant countries.

The CARDIO COVID-19–20 Registry included 3404 patients in the database, but 144 patients were excluded from the final analysis due to unresolved queries and/or data inconsistencies, resulting in 3260 patients from 44 institutions located in 14 Caribbean, Central, and South American countries (Figure 1). Of the patients included, 2059 were male (63.2%) and the median age was 61.0 (48.00, 71.00) years old. All included patients had a confirmed diagnosis of COVID-19, and 120 (3.8%) of them were healthcare workers (Table 1). Exposure type was classified as by circulation (58.8%), followed by contact (39.3%), and lastly imported (1.9%).

**Table 1:** Demographic and clinical characteristics at admission of the patients included in this cohort.


VARIABLES	N = 3260 n (%)

**Age, years (median [IQR])**	61.0 [48.0, 71.0]

**Sex**	

Male	2059 (63.2)

Female	1201 (36.8)

**Weight, kg (median [IQR])**	75.0 [67.0, 86.0]

**Sistolic blood pressure, mmHg (median [IQR])**	125.0 [112.0, 140.0]

**Diastolic blood pressure, mmHg (median [IQR])**	75.0 [67.0, 83.0]

**Heart rate, beats/min (median [IQR])**	93.0 [80.0, 106.0]

**Oxygen saturation, % (median [IQR])**	91.0 [86.0, 95.0]

**Respiratory rate, breaths/min (median [IQR])**	22.0 [19.0, 28.0]

**Fraction of inspired oxygen, % (median [IQR])**	32.0 [21.0, 85.0]

**Overweight/obesity**	1621 (49.7)

**Arterial hypertension**	1596 (49.0)

**Diabetes mellitus**	869 (26.7)

**Dyslipidemia**	451 (13.8)

**Smoking (previous/actual)**	438 (13.4)

**Organ transplant**	

Heart	9 (0.3)

Kidney	42 (1.3)

Liver	5 (0.2)

Other	4 (0.1)

**Asthma/COPD (with/without oxygen)**	287 (8.8%)

**Chronic kidney disease (with/without dialysis)**	270 (8.3)

**Coronary heart disease**	244 (7.5)

**Heart failure**	182 (5.6)

LVEF <40%	72 (48.0)

LVEF 40%–50%	36 (24.0)

LVEF >50%	42 (28.0)

Unknown	32

**Current pregnancy**	51 (1.6)

**Cancer**	139 (4.3)

**Autoimmune disease/immunosuppressant use**	127 (3.9)

**Atrial fibrillation (valvular/non valvular)**	115 (3.5)

**Stroke**	102 (3.1)

**Heart device**	54 (1.8)

Implantable cardiodefibrillator (ICD)	11 (0.3)

Cardiac resynchronization Therapy (CRT)	1 (0.0)

Pacemaker	39 (1.2)

ICD/CRT	4 (0.1)

**HIV infection**	24 (0.7)

**COVID-19 exposure type**	

Imported	54 (1.9)

Contact	1126 (39.3)

Circulation	1686 (58.8)

Unknown	394

**Health care worker**	

Yes	120 (3.8)

No	3052 (96.2)

Unknown	88

**COVID-19 signs and symptoms at admission**	

Fever	2099 (64.4)

Cough	2235 (68.6)

Fatigue	1625 (49.8)

Anorexia	634 (19.4)

Myalgias	1101 (33.8)

Diarrhea	456 (14.0)

Chest pain	446 (13.7)

Palpitations	151 (4.6)

Dyspnea	2365 (72.5)

Loss of taste	213 (6.5)

Loss of smell	223 (6.8)

Other	1132 (34.7)


The most common, preexisting comorbidities were overweight/obesity (49.7%), arterial hypertension (49.0%), diabetes mellitus (26.7%), and dyslipidemia (13.8%) (Table 1). Most common clinical signs and symptoms on admission were dyspnea (72.5%), cough (68.6%), and fever (64.4%) (Table 1). 12-lead electrocardiogram was performed in 1626 patients (49.9%), and abnormal findings included right bundle branch block (6.5%), atrial fibrillation (5.4%), left bundle branch block (3.1%), and ventricular extrasystoles (1.7%) (Table 2). Chest X-rays were performed in 3019 patients (92.6%), and abnormal findings included pulmonary infiltrates (79.9%), pulmonary congestion (18.7%), cardiomegaly (16.8%), and pleural effusion (11.0%) (Table 2). Echocardiogram was performed in 634 patients (19.4%), and abnormal findings included globally decreased systolic function (15.7%), right ventricular dysfunction (16.3%), pericardial effusion (10.9%), and inferior vena cava dilatation (15.6%) (Table 2).

**Table 2:** Diagnostic tests.


VARIABLES	n (%)

**ECG performed**	1626 (49.9)

**Rhythm, n = 1556**	

Sinus rhythm	1377 (88.5)

Ventricular extrasystole	27 (1.7)

Supraventricular extrasystole	9 (0.6)

Atrial fibrillation	84 (5.4)

Atrial flutter	7 (0.4)

Other	52 (3.3)

**QRS complex (median [IQR]), n = 1393**	90.00 [80.0, 102.0]

**Bundle Branch Block, n = 1515**	

Right	98 (6.5)

Left	47 (3.1)

**Corrected QTc interval (median [IQR]), n = 1386**	419.0 [395.0, 443.0]

**Echocardiogram performed**	634 (19.4)

**Systolic function, n = 611**	

Focally decreased	51 (8.3)

Globally decreased	96 (15.7)

Normal	444 (72.7)

**Right ventricular dysfunction, n = 589**	96 (16.3)

**Pericardial effusion, n = 625**	

Mild	57 (9.1)

Moderate	9 (1.4)

Severe	2 (0.3)

**Cardiac tamponade, n = 618**	2 (0.3)

**Severe valvular insufficiency, n = 609**	

Mitral	11 (1.8)

Aortic	3 (0.5)

Both	2 (0.3)

**Severe valvular stenosis, n = 610**	

Mitral	5 (0.8)

Aortic	12 (2.0)

Both	0 (0.0)

**Inferior vena cava dilatation, n = 576**	90 (15.6)

**LVEF (median [IQR]), n = 621**	60.0 [50.0, 65.0]

**TAPSE, mm (mediate [IQR]), n = 365**	20.0 [17.0, 23.0]

**PSAP, mm (median [IQR]), n = 357**	35.0 [28.0, 45.0]

**Strain longitudinal (median [IQR]), n = 33**	18.0 [16.0, 21.0]

**Diameter diastolic vent left, mm (median [IQR]), n = 350**	46.0 [42.0, 52.0]

**Systolic diameter vent left, mm (median [IQR]), n = 322**	30.0 [27.0, 37.0]

**Chest X-ray performed**	3019 (92.6)

**Pulmonary infiltrates**	

Unilateral	239 (7.3)

Bilateral	2366 (72.6)

**Cardiomegaly**	549 (16.8)

**Pulmonary congestion**	

Unilateral	75 (2.3)

Bilateral	533 (16.3)

**Pleural effusion**	

Unilateral	170 (5.2)

Bilateral	190 (5.8)


Laboratory tests at admission showed median leukocytes count of 8710.0mm^3^ (6310.0, 12 125.0), median hemoglobin value of 13.6gr/dl (12.1, 14.9), median platelets count of 228 500.0µl (176 000.0, 297 000.0), median creatinine levels of 0.9mg/dl (0.7, 1.3), and median NT-proBNP level of 429.0pg/ml (88.0, 2744.5) (Supplementary file 1). In contrast, laboratory tests at discharge showed differences in median lymphocytes level 1319.5mm^3^ (800.0, 1970.0), median hemoglobin value 12.1gr/dl (10.3, 13.7), median hematocrits value (36.5%), and median platelets count 283 000.0µl (202 000.0, 378 000.0) (Supplementary file 1).

The most common cardiovascular complications during hospitalization were cardiac arrhythmia (9.1%), decompensated heart failure (8.5%), pulmonary embolism (3.9%), and acute coronary syndrome (2.9%). 53.5% of patients were admitted to the ICU, a median length of stay at the ICU was 10.0 days [5.0, 18.0]. Invasive mechanical ventilation (IMV) was required in 34.2% of patients, while 15.1% required non-invasive MV (NiMV); vasopressors were needed in 27.6% of patients, inotropics in 10.3% of patients, and vasodilators in 3.7% of patients. Other procedures performed during hospitalization included central venous catheter (20.0%), cardioversion/defibrillation (3.3%), coronary arteriography (1.6%), coronary fibrinolysis/thrombolysis (1.0%), coronary angioplasty (1.0%), use of extracorporeal oxygenation membrane (ECMO) (0.9%), and intra-aortic balloon pump (0.1%) (Table 3).

**Table 3:** In-hospital interventions.


VARIABLE	N = 3260 n (%)

**Invasive Mechanical Ventilation (IMV)**	1115 (34.2)

IMV days (median [IQR])	11.0 [6.0, 19.0]

**Vasopressor use**	900 (27.6)

Vasopressor use days (median [IQR])	7.0 [3.0, 12.8]

**Inotropic use**	336 (10.3)

Inotropic use days (median [IQR])	5.0 [3.0, 10.0]

**Vasodilator use**	119 (3.7)

Vasodilator use days (median [IQR])	3.0 [2.0, 6.0]

**Central venous catheter**	651 (20.0)

Central venous catheter days (median [IQR])	12.0 [6.0, 20.0]

**Cardioversion/defibrillation**	106 (3.3)

**Coronary arteriography**	52 (1.6)

**Coronary fibrinolysis/thrombolysis**	31 (1.0)

**Coronary angioplasty**	31 (1.0)

**Extracorporeal Oxygenation Membrane (ECMO)**	29 (0.9)

ECMO days (median [IQR])	17.0 [8.0, 28.0]

**Intraaortic balloon pump (IABP)**	2 (0.1)

IABP days (median [IQR])	5.0 [4.5, 5.5]

**Intensive Care Unit (ICU)**	1745 (53.5)

ICU days (median [IQR])	10.0 [5.0, 18.0]


The most common pharmacological treatment for COVID-19 patients included corticosteroids (67.4%), thromboprophylaxis (62.0%), anticoagulation therapy (38.6%), azithromycin (33.6%), and hydroxychloroquine (21.2%) (Table 4).

**Table 4:** Pharmacological treatment for COVID-19 during hospitalization.


MEDICAMENTS*	N = 3260 n (%)

**Corticosteroids**	

Oral	154 (4.7)

Parenteral	2043 (62.7)

**Thromboprophylaxis**	2021 (62.0)

**Anticoagulation**	1257 (38.6)

**Azithromycin**	1096 (33.6)

**Hydroxychloroquine**	690 (21.2)

**Lopinovir**	234 (7.2)

**Ritonavir**	229 (7.0)

**Chloroquine**	90 (2.8)

**Plasmapheresis**	31 (1.0)

**Immunoglobulin**	23 (0.7)

**Interferon**	9 (0.3)

**Other**	1189 (36.5)


*More than one medicament can be administered to patients.

In-hospital mortality was 25.5%, being mainly non-cardiovascular, and 30-day rehospitalization and mortality after discharge was 7.3% and 2.6%, respectively (Table 5).

**Table 5:** Outcomes during hospitalization and 30-day follow up.


VARIABLE	N = 3260 n (%)

**Condition at discharge**	

Alive	2304 (70.7)

Dead	831 (25.5)

Referred	123 (3.8)

Unknown	2

**Type of death**	

Cardiovascular	172 (20.7)

Not cardiovascular	659 (79.3)

**30 DAYS FOLLOW-UP N = 2427**	

***Condition***	

Alive	1993 (97.4)

Dead	53 (2.6)

Unknown	381

**Type of death**	

Cardiovascular	11 (20.8)

Non-cardiovascular	42 (79.2)

**Rehospitalization 30 days in post-discharge**	

Yes	144 (7.3)

No	1831 (92.7)

Unknown	452


## Discussion

Our study included 3260 patients in 14 countries of LA&C with median age of 61 years, of those patients nearly 50% present are either overweight/obesity and/or arterial hypertension. Almost 80% of the patients exhibit pulmonary infiltrates identified by chest X-ray, and 11.5% of the patients had abnormal findings in ECG. Also, 53.5% of the patients were admitted to the ICU and 34.2% required IMV. There are still many unsolved questions about COVID-19 regarding its long-term outcomes and collateral impairment on infected individuals [18] and reported data until today can vary from one population to another. Thus, to be able to compare different populations and draw conclusions, each research group must share their knowledge and findings.

The SEMI-COVID-19 Registry included data from more than 25 000 patients with COVID-19 hospitalized in Spain with a mean age was 69.4 years. The most common comorbidities were hypertension (50.9%), dyslipidemia (39.7%), smoking history (30.7%), obesity (21.2%), and diabetes mellitus (19.4%) [9]. Similar information was reported in our population regarding hypertension; however, overweight/obesity and diabetes mellitus percentages were significantly higher in our study, and dyslipidemia and smoking history were lower in our study. Our findings regarding obesity are in line with the fact that obesity in LA&C was described as having the highest prevalence in the world between individuals with lower income [19,20]. Obesity is related to a subclinical inflammatory state caused by an excess of inflammatory cells in adipose tissue. A meta-analysis found a 37.6% prevalence of pre-existing obesity in COVID-19 patients with poor outcomes, also, a higher mortality rate in obese patients who had other comorbidities, the most prevalent being cardiovascular disease, hypertension, and respiratory diseases [10]. It has been described that COVID-19-related mortality in people with DM was higher in males and directly associated with age, cardiovascular, and renal complications, as well as with poorer glycemic control and higher BMI [11].

Within our cohort, we identified that 11.5% of patients presented any ECG abnormalities, the main representation was atrial fibrillation with 5.4%. The percentage and type of abnormalities varies between studies; for instance, the one conducted by Pinto-Filho et al. [21] where they identified that 21.3% of their patients present ECG abnormalities of those 11.5% presented ischemic abnormalities. In a study performed in Pakistan, 43.7% of their patients presented abnormal ECG changes, sinus tachycardia represented 67.04%, and atrial fibrillation was 23.8% [22]. Moreover, in a study performed in Ohio and Florida in the USA, 33.1% of the participants presented at least one abnormality in the ECG, of those 21% presented sinus tachycardia and 5.4% presented atrial fibrillation [23]. Among said studies, our cohort presented the lowest percentage of ECG abnormalities.

The risk of developing venous thromboembolic events in critically ill patients is higher in the presence of COVID-19, and is related to hemostatic alterations, immobility, systemic inflammatory status, mechanical ventilation, and central catheters [13]. Of our patients, 62% received thromboprophylaxis and only 1% of the patients presented coronary fibrinolysis/thrombolysis, this supports the evidence, thrombosis rates are lower in patients who received pharmacologic thromboprophylaxis [24].

Another cohort reported that 17% of COVID-19 patients required IMV, 14% non-IMV, and ECMO 2% [25]; in contrast with our study where 34.2% of patients required IMV, and 0.9% of patients ECMO. These differences could be attributable to multiple variables, and the risk factors for these therapies must be reviewed.

The main pharmacological treatment given to the patients was corticosteroids, which is one of the drugs for patients with severe COVID-19 recommended by the WHO [26]. Furthermore, another common treatment used for severe cases of COVID-19 is the thromboprophylaxis as it is reported by multiple studies [27,28].

A severe illness is characterized by diffuse alveolar damage, inflammatory infiltrates, and microvascular thrombosis. COVID-19 is associated with diffuse lung damage, and glucocorticoids may modulate inflammation-mediated lung injury and thereby reduce progression to respiratory failure and death; 67.4% of patients from our study received treatment with corticosteroids. A study conducted by the PAHO identified the decision making in LA&C, regarding the use of drugs, corticosteroids were recommended according to cumulative decision documents [29]. Moreover, in a Chilean cohort study, the early used of corticosteroids reduced mortality [30]. A randomized study showed significantly lower mortality at 28 days with dexamethasone treatment [31,32]. It has been described to have the largest benefit among patients with IMV [32].

The SEMI-COVID-19 registry reported a lower number of deaths related to COVID-19 than our registry (21% vs 25.5%). We also had a greater number of rehospitalization than reported in SEMI-COVID-19 (3.9% vs 7.3%). Deficient treatment, high comorbidities, and a poor healthcare system contributed to the higher mortality rate in LA&C. The study performed by Spina et al. [33] identified similar results in mortality and comorbidities which reflected the severe circumstances of our population that could influence those rates.

## Conclusions

We report in this LA&C population of hospitalized patients with COVID-19 that more than half of them required management in intensive care, with higher requirement of invasive mechanical ventilation and vasoactive support, resulting in a high in-hospital mortality and a considerable high 30-day post discharge rehospitalization and mortality.

More detailed analysis between comorbidities, in-hospital cardiovascular outcomes, mortality, and rehospitalization will be done in order to implement future local and regional interventions for a better approach and treatment of these population.

## Data Accessibility Statement

The authors confirm that the data supporting the findings of this study are available within the article and its supplementary materials.

## Additional File

The additional file for this article can be found as follows:

10.5334/gh.1272.s1 Supplementary file 1. Paraclinic test results.
