# Supplementary material for: Latin-American Registry of Cardiovascular Disease and COVID-19: Final Results
Source: Glob Heart. 2023 Nov 1;18(1):60. doi: 10.5334/gh.1272 (PMC10624135; doi:10.5334/gh.1272)
Supplement: Supplementary file 1. — Paraclinic test results. [file gh-18-1-1272-s1.pdf]

Supplementary file 1. Paraclinic test results

	Paraclinic test results			
**Variable**	**Paraclinics at admission n (%)**	**N**	**Paraclinics at discharge n (%)**	**N**
Complete blood count		3260		3260
0. No	22 (0.7)		365 (11.2)	
1. Yes	3238 (99.3)		2895 (88.8)	
Leukocytes_mm3 (median[IQR])	8710.0 (6310.0, 12125.0)	3235	8640.0 (6207.5, 12500.0)	2888
Lymphocytes_mm3 (median[IQR])	1030.0 (700.0, 1480.0)	3166	1319.5 (800.0, 1970.0)	2838
Hemoglobin_gr/dl (median[IQR])	13.6 (12.1, 14.9)	3196	12.1 (10.3, 13.7)	2855
Hematocrit_% (median percentage [IQR])	40.2 (36.1, 44.0)	3161	36.5 (31.3, 40.8)	2850
Platelets_ul (median[IQR])	228500.0 (176000.0, 297000.0)	3230	283000.0 (202000.0, 378000.0)	2890
Prothrombin Time_seconds (median[IQR])	13.2 (12.0, 14.6)	2540	13.2 (12.0, 15.1)	1503
Partial Thromboplastin Time_seconds (median[IQR])	31.8 (28.0, 36.8)	2352	34.0 (29.0, 42.0)	1310
INR (median[IQR])	1.1 (1.0, 1.2)	2408	1.1 (1.0, 1.3)	1391
Creatinine_mg/dl (median[IQR])	0.9 (0.7, 1.3)	3193	0.9 (0.7, 1.3)	2690
Serum Sodium_mmol/l (median[IQR])	137.0 (134.0, 140.0)	2910	138.0 (136.0, 141.0)	2494
Serum Potassium_mmol/l (median[IQR])	4.1 (3.8, 4.6)	2906	4.2 (3.8, 4.7)	2509
Blood urea nitrogen_mg/dl (median[IQR])	18.0 (12.5, 28.9)	2990	21.3 (14.3, 38.5)	2506
Lactic dehydrogenase_UI/l (median[IQR])	369.0 (269.0, 515.2)	2704	293.0 (220.0, 440.8)	1806
Aspartate Transaminase_u/l (median[IQR])	42.0 (29.0, 65.8)	2659	36.1 (24.0, 61.0)	1630
Alanine Aminotransferase_ u/l (median[IQR])	37.0 (24.0, 60.0)	2583	44.1 (27.0, 79.8)	1543
Glucose_Glycemia_mg/dl (median[IQR])	125.0 (103.0, 175.0)	2301	119.0 (95.7, 158.0)	1671
Arterial Blood Gas		3260		3260
0. No	676 (20.7)		1371 (42.1)	
1. Yes	2584 (79.3)		1889 (57.9)	
PH (median[IQR])	7.4 (7.4, 7.5)	2519	7.4 (7.4, 7.4)	1873
Oxygen Blood Pressure_mmHg (median[IQR])	71.0 (58.7, 87.0)	2576	74.0 (62.0, 88.0)	1888
Partial Pressure of Carbon Dioxide_mmHg (median[IQR])	32.3 (28.4, 37.0)	2563	37.0 (33.0, 43.1)	1886
Bicarbonate_HCO3_mmol/L (median[IQR])	21.7 (19.2, 24.0)	2477	23.5 (20.8, 26.2)	1853
PCT Test		3260		3260
0. No	455 (14.0)		1082 (33.2)	
1. Sensitive PCR	1739 (53.3)		1416 (43.4)	
2. Ultrasensitive PCR	1066 (32.7)		762 (23.4)	
Troponin Test		3260		3260
0. No	1174 (36.0)		2365 (72.5)	
1. Troponin I	376 (11.5)		99 (3.0)	

2. Troponin T	143 (4.4)		54 (1.7)	
3. Ultrasensitive Troponin I	1216 (37.3)		489 (15.0)	
4. Ultrasensitive Troponin T	351 (10.8)		253 (7.8)	
Natriuretic Peptide Test		3260		3260
0. No	2711 (83.2)		3026 (92.8)	
1. BNP	94 (2.9)		50 (1.5)	
2. NT-proBNP	455 (14.0)		184 (5.6)	
CPK_U/L (median[IQR])	104.0 (53.0, 261.0)	1281	77.0 (34.7, 256.5)	683
D-dimer_ug/mL (median[IQR])	0.8 (0.4, 1.5)	2534	0.9 (0.4, 2.0)	1663
Fibrinogen_mg/dL (median[IQR])	550.0 (436.0, 663.0)	1244	457.5 (354.0, 574.8)	766
Ferritin_ng/mL (median[IQR])	820.0 (396.0, 1481.5)	2347	759.5 (367.2, 1333.0)	1502
Sensitive PCR_mg/dl (median[IQR])	10.1 (4.5, 20.0)	1734	2.5 (0.8, 7.9)	1415
Ultrasensitive PCR_mg/dl (median[IQR])	11.6 (4.5, 22.2)	1064	2.8 (0.8, 9.0)	760
Troponin I_ng/mL (median[IQR])	0.0 (0.0, 0.1)	375	0.1 (0.0, 0.3)	99
Troponin T_ng/mL (median[IQR])	0.0 (0.0, 0.1)	143	0.0 (0.0, 0.0)	54
Ultrasensitive Troponin I_ng/mL (median[IQR])	0.0 (0.0, 0.0)	1216	0.0 (0.0, 0.1)	489
Ultrasensitive Troponin T_ng/mL (median[IQR])	0.0 (0.0, 0.0)	351	0.0 (0.0, 0.1)	253
BNP_pg/mL (median[IQR])	100.2 (40.0, 432.2)	94	80.2 (33.0, 219.9)	50
NT_proBNP_pg/mL (median[IQR])	429.0 (88.0, 2744.5)	455	427.5 (131.5, 2954.2)	184
